# An Unusual Presentation of Retrobulbar Cavernous Hemangioma in a Young Woman

**DOI:** 10.7759/cureus.17508

**Published:** 2021-08-27

**Authors:** Anupam Singh, Rakesh Panyala, Khan Shama A Irfan, Ramanuj Samanta, Barun Kumar

**Affiliations:** 1 Ophthalmology, All India Institute of Medical Sciences, Rishikesh, Rishikesh, IND; 2 Cardiology, All India Institute of Medical Sciences, Rishikesh, Rishikesh, IND

**Keywords:** orbital cavernous hemangioma, orbital exenteration, proptosis, ct-arteriography, orbital pseudotumour

## Abstract

A 38-year-old woman presented with sudden-onset painful lid swelling, proptosis and external ophthalmoplegia on the right side for 20 days, associated with loss of vision for nine days. On contrast-enhanced computed tomography (CECT), a retrobulbar mass was noted involving intraconal and extraconal spaces, extending up to the orbital foramina with enhancement and thickening of meninges. CT arteriography further revealed multiple feeding vessels from the maxillary artery. Embolization of feeding vessels followed by right orbital exenteration with primary reconstruction using forehead flap was done. This is an unusual case of orbital cavernous hemangioma (OCH) which emphasizes the importance of CT arteriography in specific cases of OCH, where routine neuroimaging may be inconclusive.

## Introduction

Orbital cavernous hemangiomas (OCHs) are one of the most common unilateral intraconal benign orbital vascular tumours [1-3]. They are slow-growing painless tumours occurring in middle-aged adults with relative sparing of vision and ocular motility [1-3]. The axial proptosis is the most common presentation of OCH. Less common symptoms of orbital pain, headache, eyelid swelling, diplopia and gaze-induced amaurosis can occur [1-3]. Bone destruction, compressive optic neuropathy and hemodynamic disturbances in surrounding soft tissues may develop due to mass effect. Observation may be reasonable if the lesion is small, asymptomatic and if no malignancy is suspected [4]. But as the natural history of cavernous hemangioma is to enlarge, excision as soon as the diagnosis is established is advisable [2]. The aim of the surgery is the total removal of the lesion but sparing the vital structures as much as possible. En bloc excision is preferable to avoid intraoperative bleeding and the risk of recurrence [2-4].

We report an unusual acute presentation of an orbital cavernous hemangioma involving both intraconal and extraconal spaces in a young woman, who presented with complete loss of vision. The diagnostic dilemma with conventional imaging, CT arteriography-guided management and the outcome are highlighted in this report.

## Case presentation

**Figure 1:**
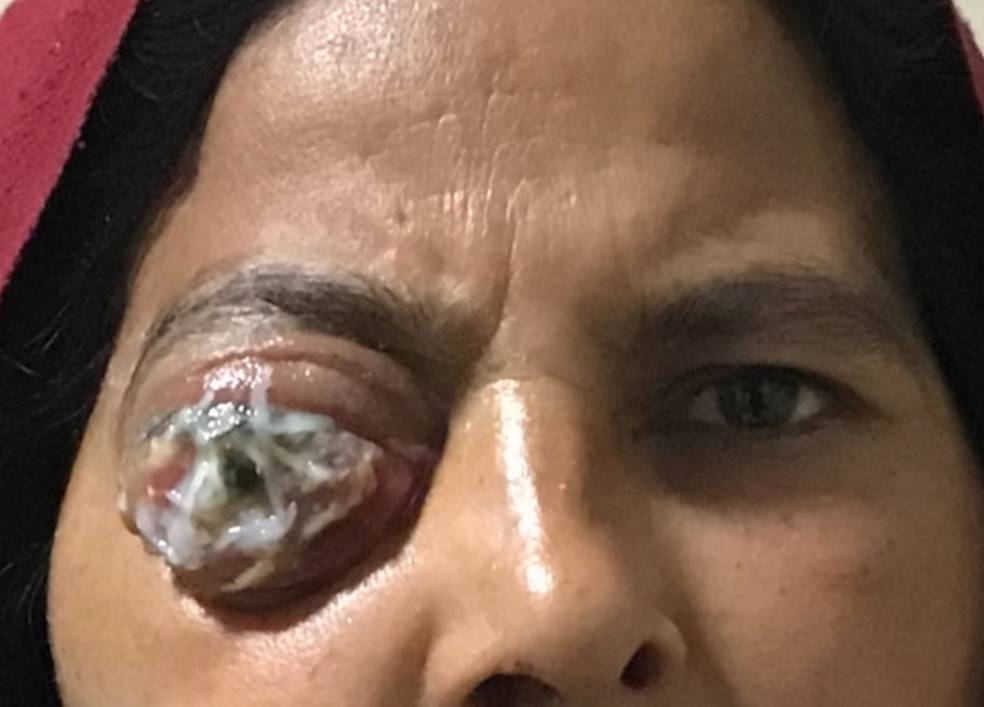
Clinical photograph of the patient showing lid swelling, outward protrusion of the eyeball, conjunctival chemosis and corneal melting at the time of presentation.

A 38-year-old woman presented with sudden-onset painful lid swelling, outward protrusion of the eyeball, inability to move the right eye and right-sided nonradiating frontal headache for 20 days, associated with loss of vision for nine days. There was no history of trauma. On examination, the right eye had no light perception and the visual acuity in the left eye was 20/20. There was marked proptosis, total ophthalmoplegia, conjunctival chemosis, corneal melting and scarring in the right eye obscuring the details of the anterior and posterior segments (Figure 1).

On examination, the left eye was unremarkable. General and systemic examination findings were normal.

**Figure 2:**
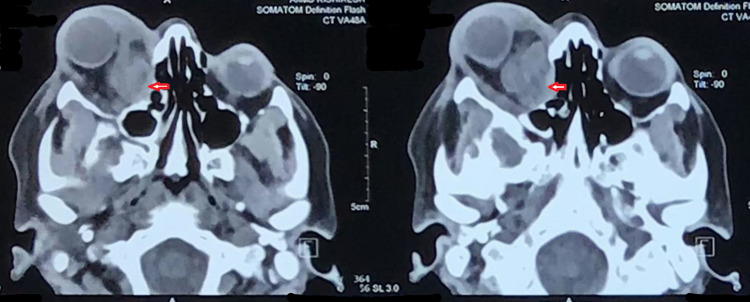
CECT of orbits and paranasal sinuses showing a retrobulbar mass with a dense calcific focus, extending to intraconal and extraconal spaces (red arrows). CECT: Contrast-enhanced computed tomography.

On orbital ultrasonography, an ill-defined, retrobulbar, lobulated heterogeneous mass was seen. Contrast-enhanced computed tomography (CECT) orbits and paranasal sinuses revealed a retrobulbar mass of size 3.5×2.8×2.7 cm with a dense calcific focus, extending to intraconal and extraconal spaces, closely abutting medial wall, and the frontal sinus, infiltrating into the optic nerve laterally and reaching up to the orbital foramina posteriorly (Figure 2, red arrows).

**Figure 3:**
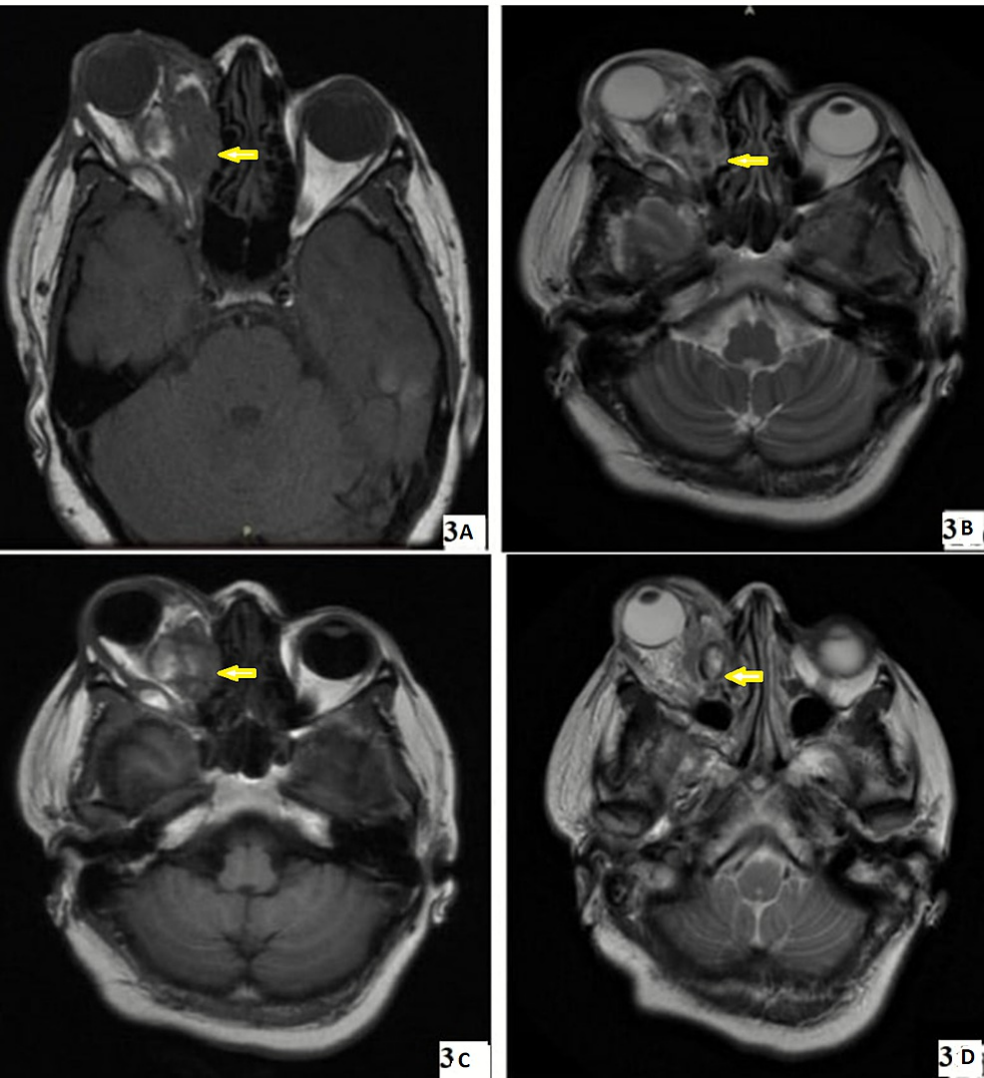
Contrast-enhanced magnetic resonance imaging of brain and orbit. (A) T1-weighted image. (B) T2-weighted image. (C) Diffusion-weighted imaging/apparent diffusion coefficient image. (D) Flair image. (A-D) Showing an ill-defined mass involving both intraconal and extraconal spaces without any evidence of intracranial extension (yellow arrows).

Contrast-enhanced magnetic resonance imaging (CE-MRI) orbit and paranasal sinus confirmed the presence of an ill-defined mass involving both intraconal and extraconal spaces. There was a ballooning of the lamina papyracea giving appearance of apparent involvement of ethmoid sinus, but the frontal sinus appeared grossly normal. MRI brain was within normal limits without any evidence of intracranial extension of the mass (Figure 3A-3D, yellow arrows).

**Figure 4:**
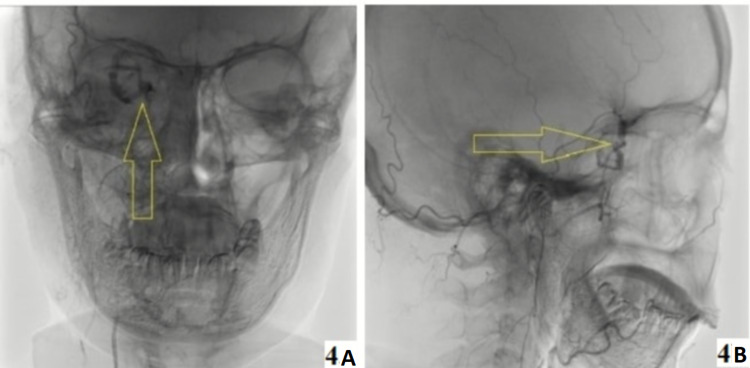
Digital subtraction angiography. (A, B) Showing feeding vessels from the maxillary artery (yellow arrows).

A provisional diagnosis of right orbital pseudotumour was made, but the patient did not respond to high-dose oral steroids even after 48 hours. On nasal endoscopy, all sinuses were clear. An endonasal biopsy was attempted after removing lamina papyracea, but it was abandoned per-operatively due to profuse bleeding, leading to hemodynamic instability. After hemodynamic stabilization, CT arteriography with digital subtraction was done which revealed multiple feeding vessels to the mass from the maxillary artery (Figure 4A-4B, yellow arrows).

**Figure 5:**
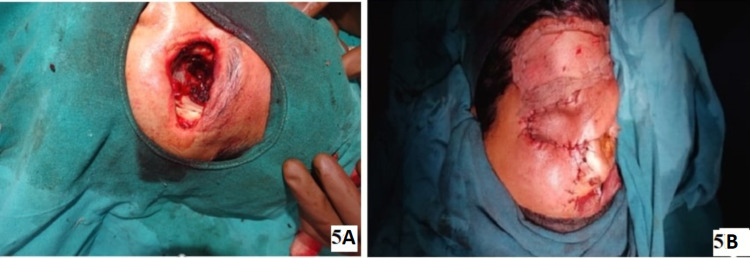
Images of orbital exenteration and primary reconstruction. (A) Exenterated socket after achieving hemostasis. (B) Orbital cavity closed with the forehead skin flap (primary closure) after the orbital exenteration.

Hence right orbital exenteration was planned after embolizing the feeding vessels. For right orbital exenteration, incision was made along the inferior orbital rim inferiorly and below the eyebrow superiorly. A 360 degree soft-tissue dissection was carried out till periosteum was visible. Then a 360 degree periosteal incision was made 5mm from the orbital rim; periosteum was elevated and lifted from the orbit. Orbital exenteration was completed, and hemostasis was achieved within 24 hours of the embolization (Figure 5A). Primary reconstruction was completed with a horizontal base axial forehead flap based on frontal branch of superficial temporal artery; eyebrow pexy was done by taking tucking sutures to the underlying tissue to prevent sagging (Figure 5B). Division and insetting of flap were done after three weeks.

**Figure 6:**
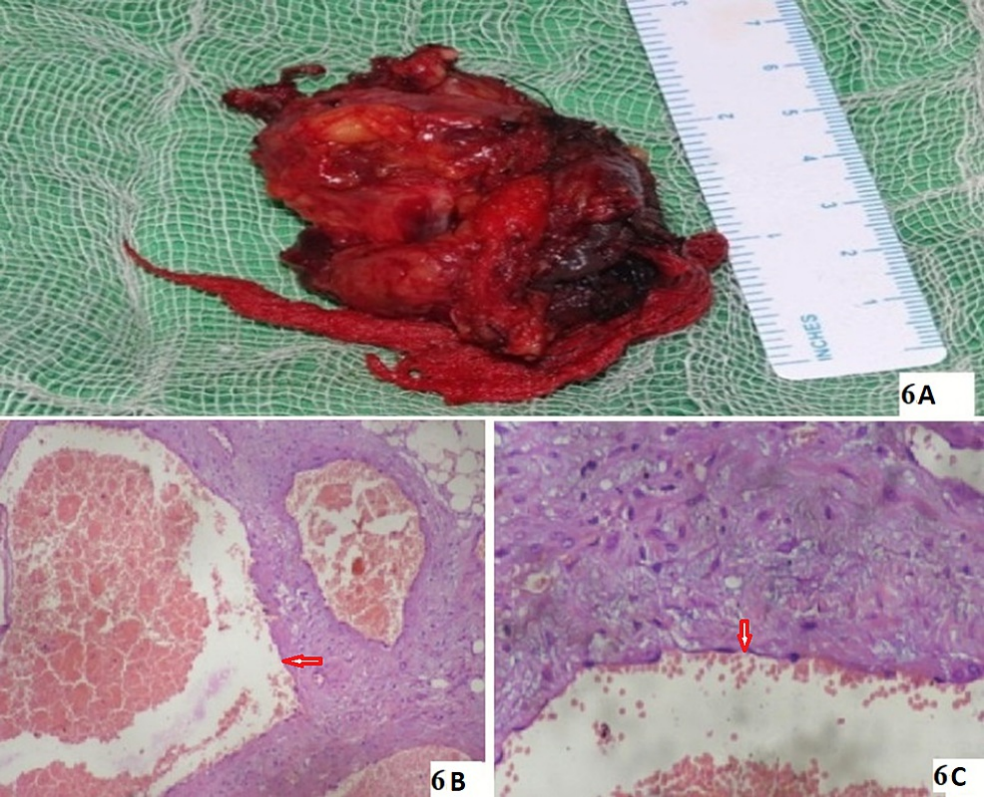
Histopathological examination of the excised mass. (A) Photomacrograph showing excised mass. (B-C) Hematoxylin and eosin staining (100× and 400× magnification) revealing large dilated vascular channels lined by flattened endothelial cells (red arrows) with an intervening fibrous interstitium suggestive of cavernous hemangioma.

On histopathological examination of the excised mass (Figure 6A), large dilated vascular channels lined by flattened endothelial cells with an intervening fibrous interstitium were seen, suggestive of OCH (Figure 6B-6C, red arrows). The histopathological findings were confirmed by two different pathologists.

**Figure 7:**
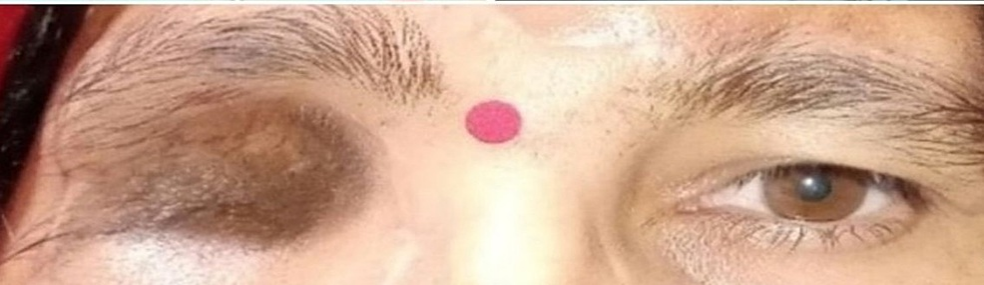
Clinical photograph of the same patient after two years of follow-up.

The patient was asymptomatic after two years with good cosmesis (Figure 7).

## Discussion

OCH is a venous malformation due to the proliferation of capillaries, the mechanism of growth being uncertain [4-6]. Histopathological examination reveals large, endothelium lined, blood-filled spaces surrounded by abundant, loosely distributed smooth muscle cells and fibrous elements [3]. Only a few cases of acute presentation have been reported in the literature due to spontaneous bleeding into the OCH [7-9].

On the basis of the hemodynamic concept, vascular malformations of the orbit have been classified into three types: type 1 (no flow) lesions having little connection to the vascular system, type 2 (venous flow) lesions with direct communication with the venous system and type 3 (arterial flow) lesions comprise arteriovenous malformations with direct anti-grade high flow through the lesion on the venous side [10]. OCH has been classified as type 3 low-flow arteriovenous malformation [10], and small feeding arteries and delayed contrast pooling have occasionally been reported using conventional prolonged digital subtraction angiography [4,11-12]. Thus, CT arteriography with digital subtraction may be of help in few selected cases.

CECT of orbit usually reveals a well-demarcated, relatively homogenous, encapsulated oval mass, which is typically located in the intraconal space or the lateral part of the middle third of the orbit [2,4]. MRI provides superior image resolution and better localization [2]. Normal and pathological intraorbital fine vascular anatomy is appropriately revealed by CT arteriography, which is not revealed by intravenously injected contrast material [4,11-12]. Therefore, it is useful for more appropriate preoperative embolization if required.

CT arteriography leads to the exact localization of tumour mass and its relationship to fine arteries and veins, extraocular muscles and the optic nerve. Thus, it is a guide for the safe manipulation of important structures during surgery [13].

In our patient, neither CECT nor CEMRI detected the possibility of the vascular tumour and was suggestive of the provisional diagnosis of orbital pseudotumour, which leads to unexpected bleeding during the diagnostic endoscopic biopsy.

As the natural history of cavernous hemangioma is to enlarge with time, most authors advise excision as soon as the diagnosis is established [2]. The aim of the surgery is total removal of the lesion, sparing the neurovascular structures as much as possible. The surgical options are direct orbitotomy, transnasal endoscopic removal, transcranial approach or orbital exenteration according to the location of the tumour [2,4,14-15]. En bloc excision is preferable to avoid intraoperative bleeding and the risk of residue and recurrence [2,4,15-16].

## Conclusions

OCH can present with sudden-onset proptosis, ophthalmoplegia, conjunctival chemosis, corneal melting and loss of vision. CECT and CEMRI may not detect the possibility of a vascular tumour or OCH in all cases. CT arteriography and embolization before surgical excision may be of great help in few suspected cases because of the high risk of per-operative bleeding. A multidisciplinary team approach may achieve favourable structural and visual outcomes, although vision may not be salvaged in advanced cases.
